# Supplementary material for: Intratumoral Heterogeneity Promotes Collective Cancer Invasion through NOTCH1 Variation
Source: Cells. 2021 Nov 9;10(11):3084. doi: 10.3390/cells10113084 (PMC8619970; doi:10.3390/cells10113084)
Supplement: Supplementary file 1 [file cells-10-03084-s001.zip › Cells-1335530-Supplementary.pdf]

Supplementary Information

Intratumoral Heterogeneity Promotes Collective Cancer Invasion Through NOTCH1 Variation

Peter Torab,[1] Yue Yan,[2] Mona Ahmed,[2] Hironobu Yamashita,[3] Joshua I. Warrick,[3,4] Jay D. Raman,[4] David J. DeGraff,[3,4,5] and Pak Kin Wong[1,2,4,*]

[1]Department of Mechanical Engineering, The Pennsylvania State University, University Park, PA 16802, USA

[2]Department of Biomedical Engineering, The Pennsylvania State University, University Park, PA 16802, USA

[3]Department of Pathology and Laboratory Medicine, The Pennsylvania State University, Hershey PA 17033, USA

[4]Department of Surgery, Penn State Health Milton S. Hershey Medical Center, Hershey, PA 17033, USA

[5]Department of Biochemistry and Molecular Biology, The Pennsylvania State University, Hershey, PA 17033, USA

* To whom correspondence should be addressed: pak@engr.psu.edu

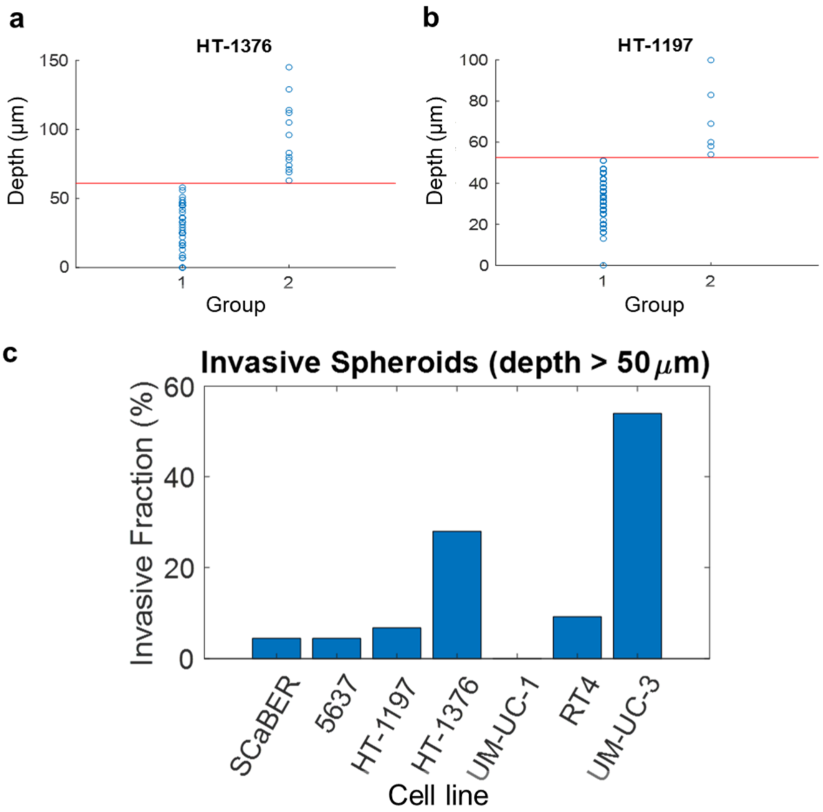

Figure S1. Clustering analysis defines the invasive fraction of microtumors in the single layer invasion model. (a-b) K-means clustering results for invasive microtumor depth in the single layer invasion model, 72 hours after seeding (k=2). In both cell line, the threshold value was determined to be approximately 50 µm. (c) Fraction of microtumors that invade Matrigel more than 50 µm. SCaBER and RT4 represent the least invasive cell lines in the basal and luminal subtypes, respectively. In addition to the luminal and basal cell lines, UM-UC-3, which is a highly invasive bladder cancer cell line and represents the neuroendocrine-like subtype, was included as a reference. Data are obtained from 3 independent experiments (n > 50 for each case, Chi-square test, ****p<0.0001).

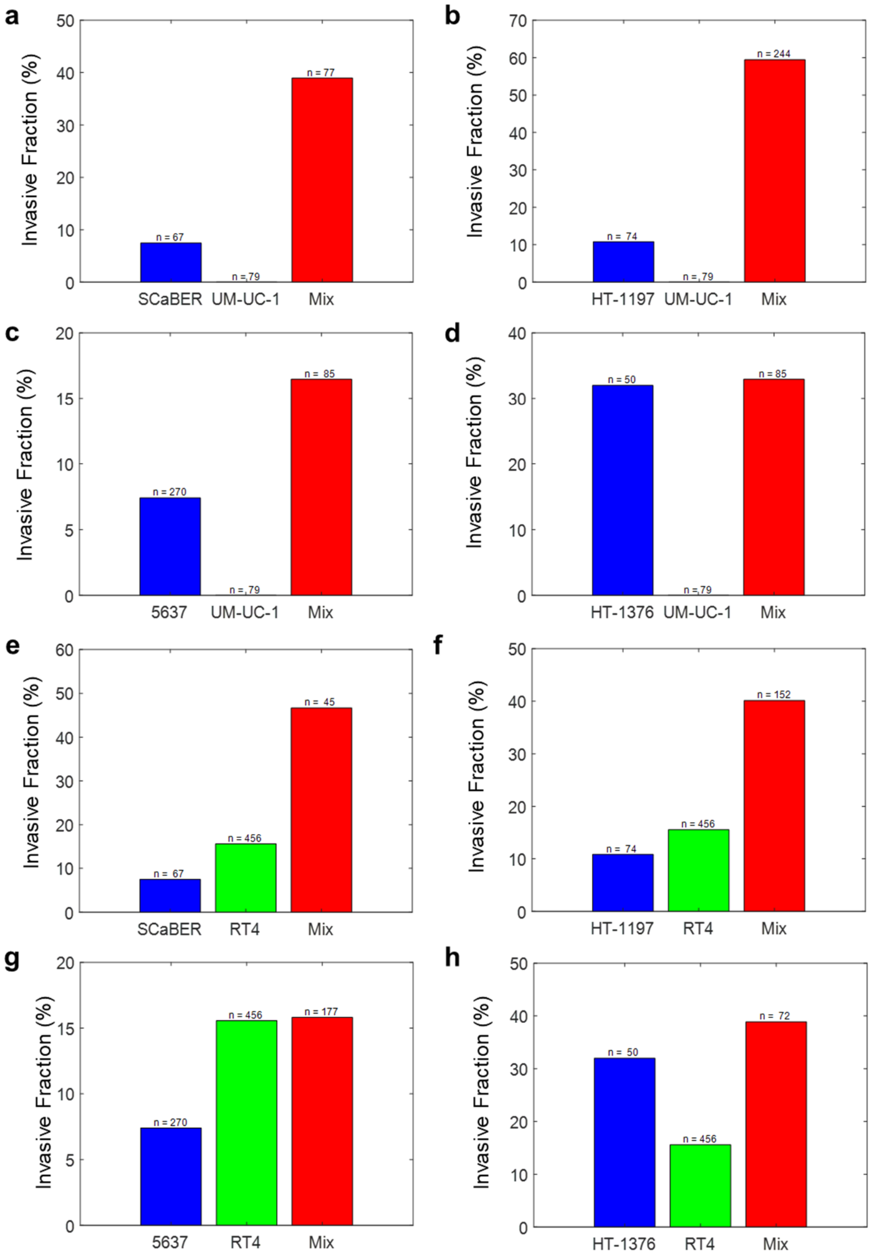

Figure S2. Co-culture enhances invasiveness of microtumors compared to individual cell lines. (a-h) Invasive fraction of mixed microtumors vs microtumors formed by individual cells lines. Data are obtained from 3 independent experiments. *n* is indicated in the chart (n>45 for each case, Fisher's exact test, ****p<0.0001 a, b, d, e, f, and h, ***p<0.001 for c, and **p<0.01 for g).

a

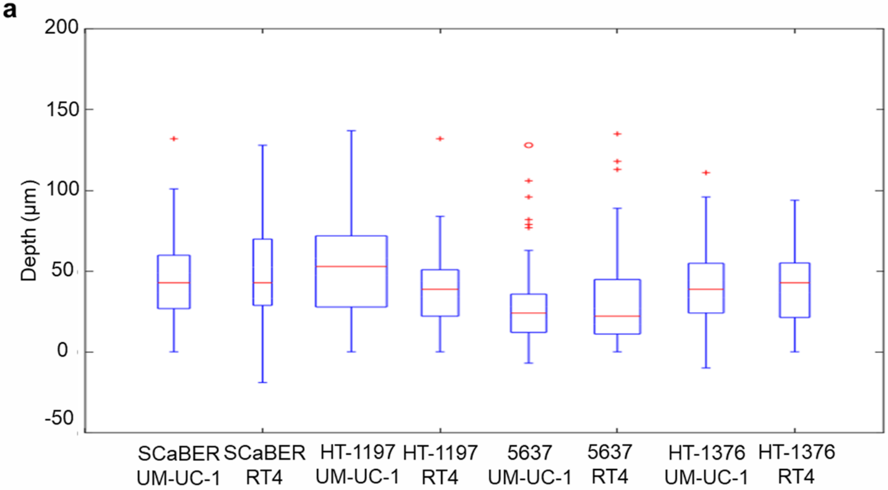

b

Group	SCaBER RT4	HT-1197 UM-UC-1	HT-1197 RT4	5637 UM-UC-1	5637 RT4	HT-1376 UM-UC-1	HT-1376 RT4
SCaBER UM-UC-1	0.9519	0.3760	0.6645	0.0025	0.0047	0.9978	0.9886
SCaBER RT4	-	0.9996	0.1478	0.0002	0.0004	0.6797	0.5801
HT-1197 UM-UC-1		-	0.0004	0.0000	0.0000	0.0519	0.0387
HT-1197 RT4			-	0.3083	0.4739	0.9593	0.9929
5637 UM-UC-1				-	0.9999	0.0207	0.0644
5637 RT4					-	0.0383	0.1143
HT-1376 UM-UC-1						-	1.0000

Figure S3. (a) Comparison of invasion depth of mixed microtumors of luminal and basal cells. Data represent mean ± SEM (n>45 for each case, ****p <0.0001). (b) p-values for pairwise comparison. Values below 0.05 are highlighted red. Kruskal-Wallis ANOVA test with Tukey-Kramer post-hoc test.

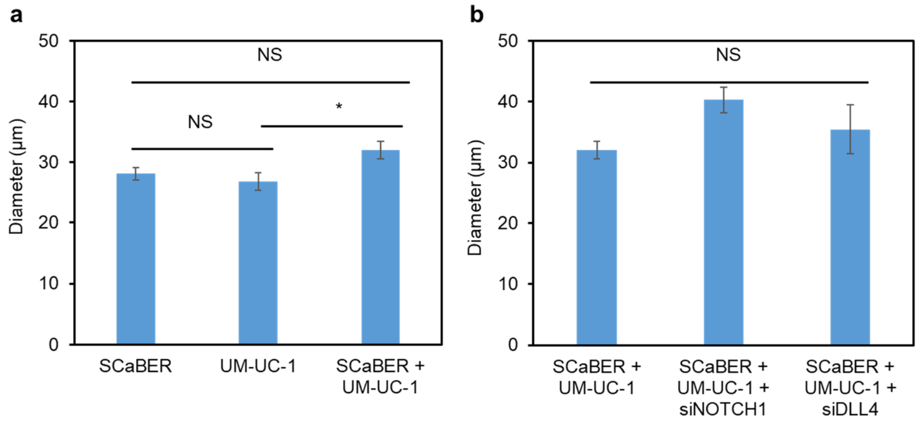

Figure S4. Size distributions of homogeneous and heterogeneous microtumors. (a) Both individual cell lines and SCaBER-UM-UC-1 co-culture formed 3D microtumors approximately 30 µm in diameter. Data are representative of 3 independent experiments. (b) NOTCH1 and DLL4 siRNA did not have an effect on the dimeter of the SCaBER-UM-UC-1 mixed microtumors. Data represent mean ± SEM (n>45, NS p>0.05, *p<0.05, Kruskal-Wallis ANOVA test with Tukey's honestly significant difference test).

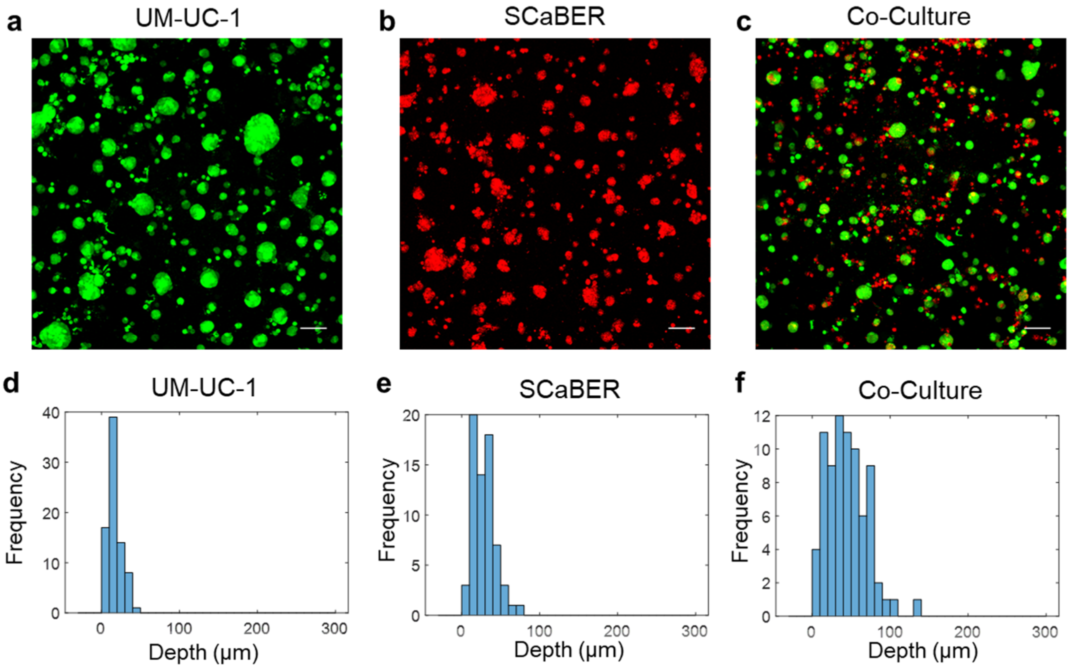

Figure S5. SCaBER and UM-UC-1 invasion of Matrigel. (a-c) Vertical projection views of the single layer invasion model. Scale bars, 100 μm. (d-e) Distribution of depth of microtumor invasion into Matrigel. Data are representative of three independent experiments.

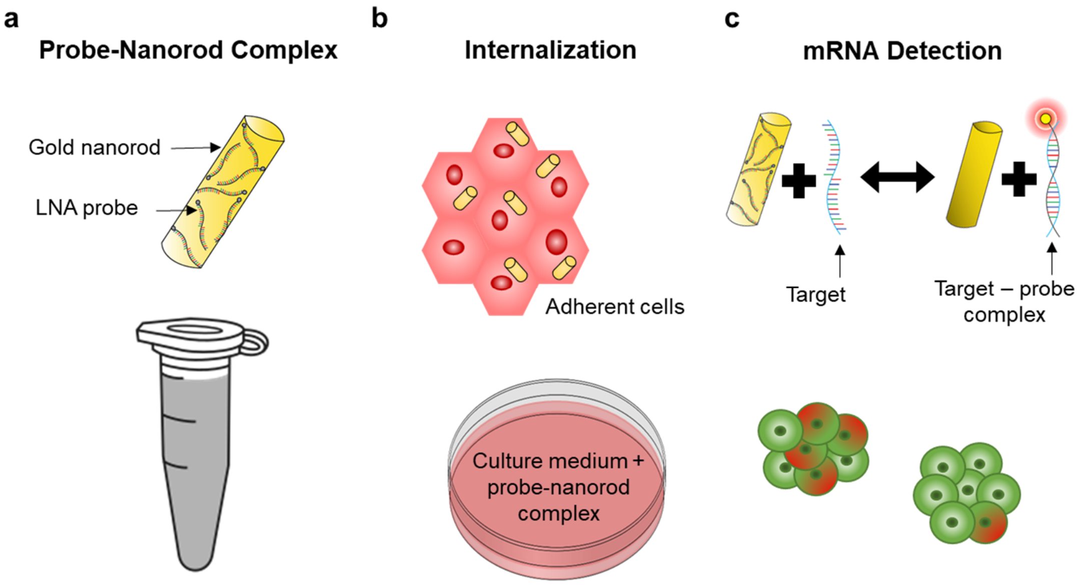

Figure S6. Live cell biosensors for measuring NOTCH1 and DLL4 expression in 3D microtumors. (a) Schematic of gold nanorod-locked nucleic acid (GNR-LNA) biosensors. (b) The biosensors are internalized into the cells before microtumor formation to ensure uniform loading among the cells. (c) The cells are then self-assembled on ECM mimicking gel to form 3D microtumors.

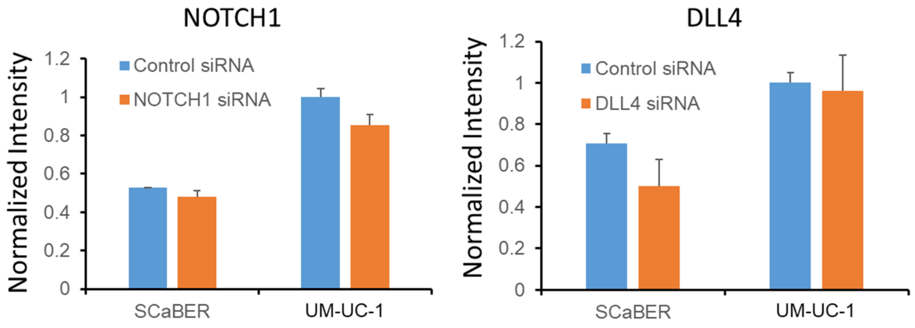

Figure S7. Transient knockdown efficiency of NOTCH1 and DLL4 siRNA. Normalized intensity of NOTCH1 and DLL4 biosensors in SCaBER and UM-UC-1 3D microtumors. Data represent mean ± SEM and are determined from at least 12 microtumors for each case.

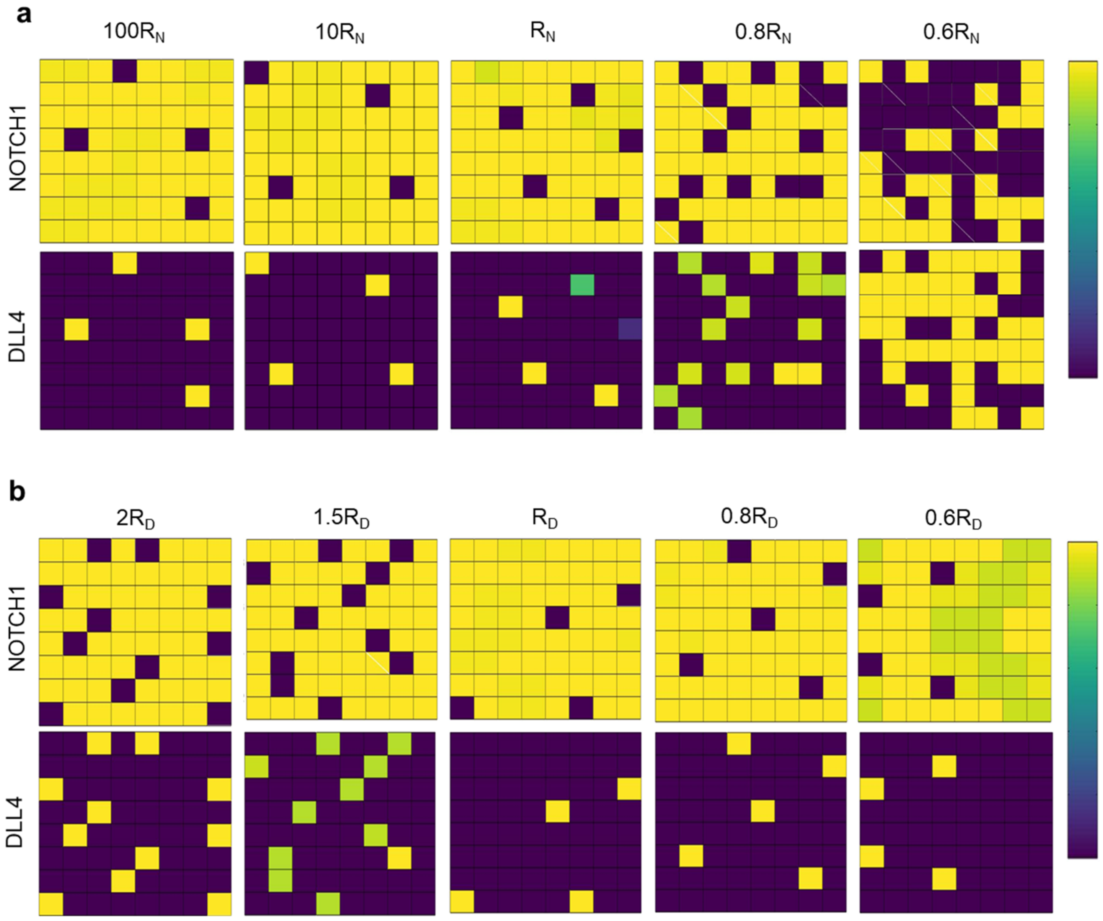

Figure S8. Computation modeling of NOTCH1 and DLL4. (a-b) Agent-based model for studying the effects of NOTCH1 and DLL4 on pattern formation. Sensitivity analysis of (a) NOTCH1 production rates R_N and (b) DLL4 production rate R_D for evaluating the effects of NOTCH1 and DLL4 production rates on the spatial pattern. The base values of R_N and R_D were 0.01.

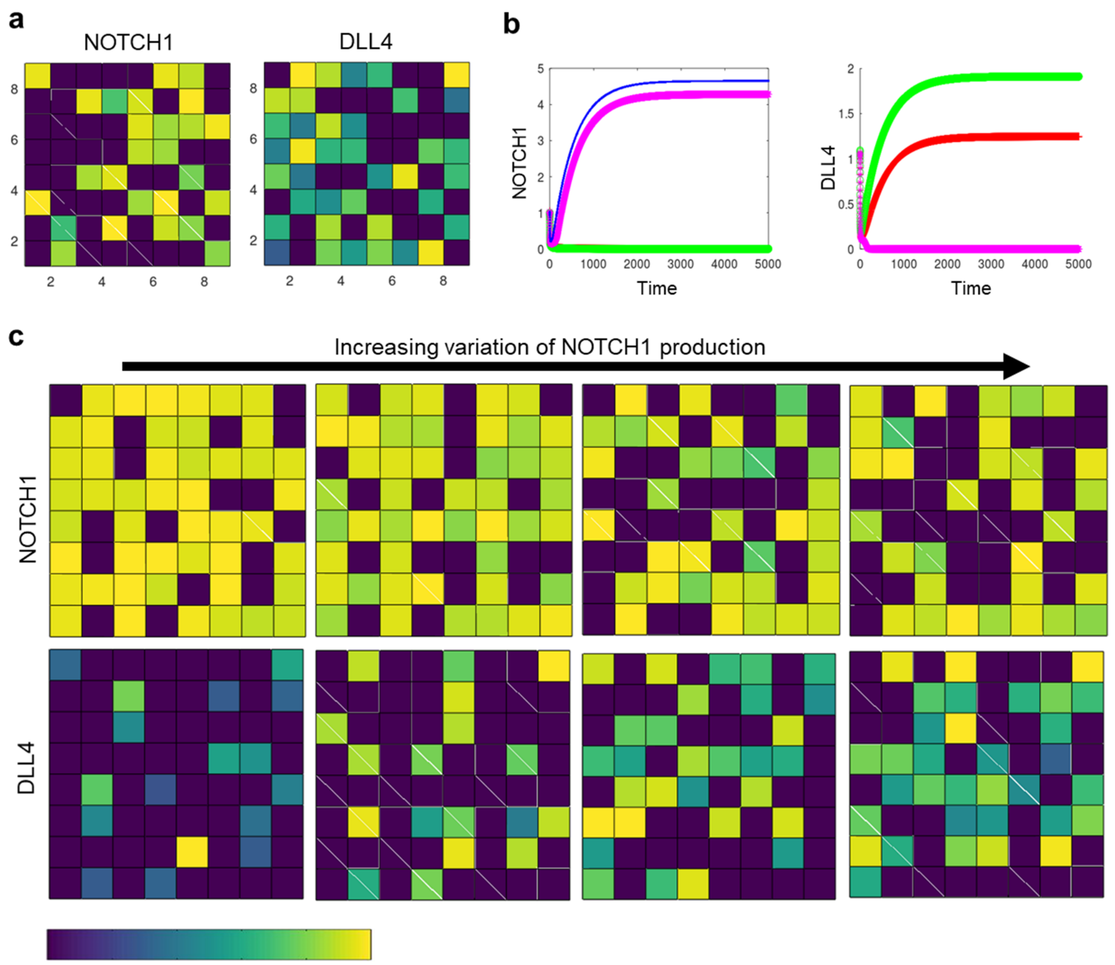

Figure S9. Computation modeling of DLL4 expression with variation of NOTCH1 production rate. (a) Introduction of NOTCH1 variation resulted in cluster of DLL4 expressing cells. (b) Tracking of NOTCH1 and DLL4 activities in representative cells committed to NOTCH1 or DLL4 phenotypes. (c) Effects of increasing NOTCH1 variation on DLL4 expression within the microtumor. The color bar shows the level of NOTCH1 and DLL4 in arbitrary units.

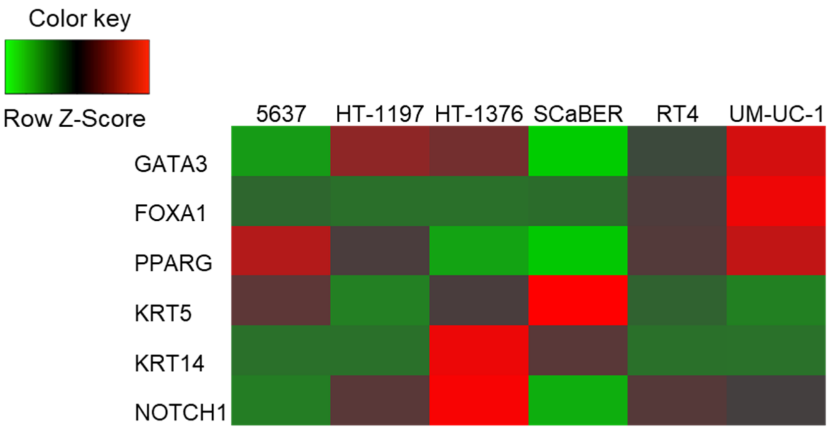

Figure S10. Comparison of cancer cell line encyclopedia RNA-seq data for luminal and basal cell lines. RT4 and UM-UC-1 express typical markers of luminal cells (GATA3, FOXA1, and PPARG) while 5673, HT-1197, HT-1376, and SCaBER express typical markers of basal cells (KRT5 and KTR14). These cell lines, such as the UM-UC-1 and SCaBER pair, express distinct levels of NOTCH1 at the transcriptional level.

Supplementary Tables

Table S1. Classification of cell lines based on the consensus classification of MIBC classifier

Cell line	Classification	cor_pval	Separation Level	LumP	LumNS	LumU	Stroma-rich	Ba/Sq	NE-like
5637	Ba/Sq	7.2123E-38	0.893348545	0.243010277	0.148407183	0.214797702	0.203685458	0.450765564	0.25256585
HT-1197	Ba/Sq	1.14627E-29	0.760629435	0.317086385	0.254977757	0.315632216	0.258204165	0.401352433	0.265503397
HT-1376	Ba/Sq	1.73258E-42	0.684089503	0.353514899	0.271735141	0.322733231	0.264430973	0.47538817	0.216371779
SCaBER	Ba/Sq	3.98863E-49	0.842180346	0.203077725	0.092416082	0.128169596	0.163858607	0.507588991	0.127380803
RT4	LumP	2.70353E-71	0.487301754	0.595295829	0.465008717	0.492565423	0.303953405	0.264866974	0.168592853
UM-UC-1	LumP	1.29073E-57	0.419950298	0.544314462	0.426945277	0.440096918	0.393601022	0.497786756	0.198979927

Table S2. Chi-square analysis and Fisher's exact test of data in Figure 2e. The conditions include SCaBER homogeneous culture, UM-UC-1 homogeneous culture, and SCaBER-UM-UC-1 co-culture

	SCaBER	UM-UC-1	Co-Culture
Medium interface	44 (46.06) [0.09]	82 (64.12) [4.99]	29 (44.82) [5.58]
Matrigel	22 (15.16) [3.09]	16 (21.10) [1.23]	13 (14.75) [0.21]
Collagen I interface	4 (7.13) [1.38]	3 (9.93) [4.83]	17 (6.94) [14.58]
Collagen I	4 (5.65) [0.48]	2 (7.86) [4.37]	13 (5.49) [10.25]

Chi-Square = 51.0895, Degrees of Freedom = 6, p = 2.62803e-9
Values indicate: observed spheroids (expected number) [chi-square statistic]
Pairwise Fisher's exact test. *$p<0.05$ SCaBER vs UM-UC-1, ****$p<0.0001$ SCaBER vs co-culture, ****$p<0.0001$ UM-UC-1 vs co-culture.

Table S3. Chi-square analysis and Fisher's exact test of data in Figure 4c. The conditions include SCaBER-UM-UC-1 co-culture, SCaBER-UM-UC-1 (DLL4 siRNA), SCaBER (DLL4 siRNA)-UM-UC-1, and SCaBER (DLL4 siRNA)-UM-UC-1 (DLL4 siRNA)

	Co-Culture (Control siRNA)	UM-UC-1 KD	SCaBER KD	Both KD
Medium interface	46 (54.84) [1.423]	29 (25.18) [0.57]	21 (20.15) [0.036]	26 (21.83) [0.80]
Matrigel	43 (38.21) [0.60]	16 (17.5) [0.14]	13 (14.04) [15.21]	13 (15.21) [0.32]
Collagen I interface	9 (4.94) [3.33]	0 (2.27) [2.27]	2 (1.81) [0.019]	0 (1.97) [1.97]
Collagen I	0 (0)	0 (0)	0 (0)	0 (0)

Values indicate: observed spheroids (expected number) [chi-square statistic]
Pairwise Fisher's exact test. *$p<0.05$ co-culture vs UM-UC-1 KD, NS co-culture vs SCaBER KD, *$p<0.05$ co-culture vs both KD

Table S4. Chi-square analysis and Fisher's exact test of data in Figure 4d. The conditions include SCaBER-UM-UC-1 co-culture, SCaBER-UM-UC-1 (NOTCH1 siRNA), SCaBER (NOTCH1 siRNA)-UM-UC-1, and SCaBER (NOTCH1 siRNA)-UM-UC-1 (NOTCH1 siRNA)

	Co-Culture (Control siRNA)	UM-UC-1 KD	SCaBER KD	Both KD
Medium interface	46 (42.37) [0.31]	13 (14.27) [0.11]	9 (8.65) [0.014]	12 (14.70) [0.50]
Matrigel	43 (37.61) [0.77]	11 (12.66) [0.22]	4 (7.67) [[1.76]	13 (13.05) [0.00]
Collagen I interface	9 (7.41) [0.34]	0 (2.50) [2.50]	0 (1.51) [1.51]	5 (2.57) [2.29]
Collagen I	0 (10.59) [10.59]	9 (3.57) [8.27]	7 (2.16) [10.882]	4 (3.67) [0.029]

Chi-Square = 40.04, Degrees of Freedom = 9, p = 0.00000746168
Values indicate: observed spheroids (expected number) [chi-square statistic]
Pairwise Fisher's exact test. ****$p<0.0001$ co-culture vs UM-UC-1 KD, ****$p<0.0001$ co-culture vs SCaBER KD, ***$p<0.001$ co-culture vs both KD

Table S5. Chi-square analysis and Fisher's exact test of data in Figure 5b. The conditions include UM-UC-1 WT, FOXA-1 KO, and co-culture

	Wild Type	FOXA1-KO	Co-Culture
Medium interface	82 (74.30) [0.80]	32 (31.02) [0.03]	18 (26.69) [2.83]
Matrigel	16 (16.89) [0.05]	3 (7.05) [2.33]	11 (6.07) [4.01]
Collagen I interface	3 (6.19) [1.64]	6 (2.58) [4.51]	2 (2.22) [0.02]
Collagen I	2 (5.63) [2.34]	2 (2.35) [0.05]	6 (2.02) [7.83]

Chi-Square = 26.4441, Degrees of Freedom = 6, p = 0.000183981

Values indicate: observed spheroids (expected number) [chi-square statistic]

Pairwise Fisher's exact test. *$p<0.05$ WT vs KO, ****$p<0.0001$ WT-co-culture, ***$p<0.001$ KO vs co-culture

Supplementary Movies

Movie S1. Heterogeneous microtumors in 3D microenvironment. Heterogeneous microtumors formed by bladder cancer cells of luminal papillary (UM-UC-1) and basal/squamous (SCaBER) subtypes. The Red: SCaBER; Green: UM-UC-1. 3D microtumors are approximately 50 μm in diameter.

Movie S2. Heterogeneous microtumors formed by bladder cancer cells of luminal papillary (UM-UC-1) and basal/squamous (SCaBER) subtypes. The Red: SCaBER; Green: UM-UC-1. 3D microtumors are approximately 50 μm in diameter.

Movie S3. Heterogeneous microtumors formed by bladder cancer cells of luminal papillary (UM-UC-1) and basal/squamous (SCaBER) subtypes. The Red: SCaBER; Green: UM-UC-1. 3D microtumors are approximately 50 μm in diameter.
